# The Neurotherapeutic Arsenal in *Cannabis sativa*: Insights into Anti-Neuroinflammatory and Neuroprotective Activity and Potential Entourage Effects

**DOI:** 10.3390/molecules29020410

**Published:** 2024-01-15

**Authors:** Ahmad K. Al-Khazaleh, Xian Zhou, Deep Jyoti Bhuyan, Gerald W. Münch, Elaf Adel Al-Dalabeeh, Kayla Jaye, Dennis Chang

**Affiliations:** 1 NICM Health Research Institute, Western Sydney University, Penrith, NSW 2751, Australia; p.zhou@westernsydney.edu.au (X.Z.); d.bhuyan@westernsydney.edu.au (D.J.B.); g.muench@westernsydney.edu.au (G.W.M.); 19255718@student.westernsydney.edu.au (K.J.); 2 School of Science, Western Sydney University, Penrith, NSW 2751, Australia; 3 Pharmacology Unit, School of Medicine, Western Sydney University, Penrith, NSW 2751, Australia; 4 Department of Biological Sciences, School of Science, University of Jordan, Amman 11942, Jordan; elaf.ald@yahoo.com

**Keywords:** *Cannabis sativa*, cannabinoids, entourage effects, flavonoids, neuroinflammatory, neuroprotective diseases, phytochemicals, synergistic effects, terpenes

## Abstract

Cannabis, renowned for its historical medicinal use, harbours various bioactive compounds—cannabinoids, terpenes, and flavonoids. While major cannabinoids like delta-9-tetrahydrocannabinol (THC) and cannabidiol (CBD) have received extensive scrutiny for their pharmacological properties, emerging evidence underscores the collaborative interactions among these constituents, suggesting a collective therapeutic potential. This comprehensive review explores the intricate relationships and synergies between cannabinoids, terpenes, and flavonoids in cannabis. Cannabinoids, pivotal in cannabis’s bioactivity, exhibit well-documented analgesic, anti-inflammatory, and neuroprotective effects. Terpenes, aromatic compounds imbuing distinct flavours, not only contribute to cannabis’s sensory profile but also modulate cannabinoid effects through diverse molecular mechanisms. Flavonoids, another cannabis component, demonstrate anti-inflammatory, antioxidant, and neuroprotective properties, particularly relevant to neuroinflammation. The entourage hypothesis posits that combined cannabinoid, terpene, and flavonoid action yields synergistic or additive effects, surpassing individual compound efficacy. Recognizing the nuanced interactions is crucial for unravelling cannabis’s complete therapeutic potential. Tailoring treatments based on the holistic composition of cannabis strains allows optimization of therapeutic outcomes while minimizing potential side effects. This review underscores the imperative to delve into the intricate roles of cannabinoids, terpenes, and flavonoids, offering promising prospects for innovative therapeutic interventions and advocating continued research to unlock cannabis’s full therapeutic potential within the realm of natural plant-based medicine.

## 1. Introduction

Plant-derived compounds have emerged as promising neuroprotective agents due to their diverse mechanisms of action and potential therapeutic effects. Various studies have highlighted the neuroprotective abilities of these compounds against a range of neurodegenerative disorders, such as Alzheimer’s, Parkinson’s, and Huntington’s diseases [1,2,3,4]. Additionally, plant-derived neuroprotective agents have been reported to exhibit antioxidant, anti-inflammatory, anti-aggregation, anti-cholinesterase, and anti-apoptotic properties, all of which are important in preserving the structure and function of neurons [2,3]. Therefore, the diverse mechanisms of action and the potential of plant-derived compounds to mitigate neurodegenerative processes make them promising candidates for developing neuroprotective therapies.

Cannabis, known by various names such as marijuana, ganja, hashish, pot, and hemp, is an ancient plant cultivated and exploited for its various properties. It is a versatile plant, being used as a fibre source, food ingredient, and medicinal substance [5]. This annual flowering herb can be classified into three primary species: *Cannabis sativa*, which is taller and more fibrous, and *Cannabis indica*, which is shorter and more psychoactive. Both species exist in both wild and cultivated forms. Additionally, some taxonomists propose including a third putative species, *Cannabis ruderalis*, which is solely wild [5].

Cannabis is a genus within the Cannabaceae plant family, including hops. A defining characteristic of all Cannabis plants is the presence of secondary substances called cannabinoids or phytocannabinoids [6]. At the same time, the genus comprises three species, *C. sativa*, *C. ruderalis*, and *C. indica. C. sativa* is the most extensively studied species in terms of its medicinal potential, unlike *C. ruderalis* and *C. indica*, which require further elucidation regarding their therapeutic properties [6].

*C. sativa* holds significant value as a medicinal plant and has garnered increasing interest in the research and manufacturing sectors. To date, over 150 cannabinoids and numerous other compounds, including terpenoids, flavonoids, and alkaloids, have been identified in *C. sativa* [7,8,9]. Many traditional medicinal uses of *C. sativa* have been studied [9]. Furthermore, cannabis has historically been employed in treating various ailments, including pain, inflammation, and mental illnesses. However, it is important to note that discrepancies in terminology between historical texts and modern scientific literature, as well as potential nuances lost in translation between Chinese and English, may exist [9].

Hemp refers to the *C. sativa* plant cultivated for industrial, agricultural, and medicinal purposes. Unlike its cousin, marijuana, hemp contains very low levels of tetrahydrocannabinol (THC), the psychoactive compound responsible for the “high” associated with marijuana. Moreover, hemp in many countries is legally defined as having a THC content of 0.3% or less on a dry-weight basis [10]. Hemp seeds are recognized as a nutritionally dense superfood, boasting essential fatty acids, proteins, fibre, and a range of vitamins and minerals [11]. Rich in omega-3 fatty acids, particularly alpha-linolenic acid, hemp seeds and oil contribute to heart health, aid brain function, and display anti-inflammatory properties [12,13]. A notable attribute is hemp seeds offering all nine essential amino acids, making it a complete protein source crucial for bodily functions, a feature particularly beneficial for vegetarians and vegans [14,15]. Hemp products, including flour, contribute to dietary fibre intake, promoting digestive health and regulating blood sugar [14,16]. The presence of cannabinoids and terpenes in hemp, with a focus on the non-psychoactive cannabinoid cannabidiol (CBD), showcases anti-inflammatory and potential analgesic effects, supporting its consideration for pain management [17]. Moreover, hemp seed oil’s fatty acid richness makes it a moisturizing option for skin health, potentially alleviating dry skin and conditions like eczema [10,11]. The omega-3 fatty acids in hemp may decrease the risk of heart disease, improve cholesterol levels, and support overall heart function [14].

The historical trajectory of hemp, from the mid-20th century to the present day, reveals a complex interplay of regulatory challenges, socio-political dynamics, and evolving scientific perspectives [18]. During the mid-20th century, hemp encountered formidable regulatory obstacles, particularly in the United States, where the classification of cannabis as a Schedule I substance in the 1970s impeded cultivation and research efforts, blurring the distinction between industrial hemp and marijuana [18]. This era witnessed a decline in hemp cultivation due to restrictive regulatory environments and the association of hemp with its psychoactive counterpart, marijuana [18]. However, the ensuing decades marked a transformative period characterized by renewed interest and legislative changes. The introduction of the U.S. Farm Bill in 2014 and subsequent updates facilitated pilot programs and research initiatives, fostering a climate conducive to legal hemp cultivation [19]. Scientific exploration flourished with advances in analytical techniques and a relaxation of legal constraints, prompting an increased focus on understanding the phytochemistry of hemp, including cannabinoids like CBD and THC and exploring the potential therapeutic applications of these compounds [20]. The entourage effect gained prominence during this period, underscoring the synergistic interactions among cannabinoids, terpenes, and other phytochemicals in hemp [17]. The emergence of CBD as a non-psychoactive cannabinoid with purported anti-inflammatory, analgesic, and neuroprotective properties fuelled a significant boom in both research and commercial interest, contributing to the burgeoning market for CBD products [5]. Diverse hemp cultivars tailored for fibre, seed production, or high cannabinoid content gained acceptance, reflecting an expanding understanding of hemp’s potential applications [21]. Global expansion ensued, with many countries embracing or relaxing restrictions on hemp cultivation, fostering an international exchange of knowledge and practices [22]. Furthermore, environmental considerations highlighted hemp’s sustainability, positioning it as a low-impact, high-yield crop suitable for diverse climates [11]. In summation, the journey of hemp from regulatory constraints to global acceptance underscores a dynamic and multifaceted evolution influenced by regulatory shifts, scientific advancements, and changing societal attitudes.

The taxonomic identification and classification of *C. sativa* have been intricate endeavours shaped by historical curiosity and scientific inquiry. The plant’s substantial morphological and genetic variability has resulted in recognising distinct forms or subspecies, including *C. sativa*, *C. indica*, and *C. ruderalis* [21]. This diversity extends to phytochemical composition, encompassing variations in cannabinoids (such as THC and CBD), terpenes, and other compounds, contributing to the array of effects and applications associated with different hemp types [23]. Cultivation practices tailored for industrial, medicinal, or recreational purposes have further influenced the development of specific cultivars, emphasising traits such as fibre production, seed quality, or cannabinoid content [24]. Acceptance of particular hemp types is intricately tied to regulatory considerations, where legal definitions, especially concerning THC content, play a pivotal role [22]. Standardisation efforts in the hemp industry, particularly for non-drug applications like textiles and paper, have driven acceptance based on low-THC content criteria [21]. The rise of CBD-dominant hemp strains for medicinal purposes underscores a contemporary trend, reflecting evolving market demands and the growing interest in the therapeutic potential of cannabinoids [5]. International harmonisation efforts in hemp regulations contribute to the acceptance of specific types globally [25]. Furthermore, economic considerations, such as market preferences for fibre quality or high CBD content, shape the acceptance landscape [26]. Moreover, acknowledging diverse *C. sativa* types is intricately linked to botanical characteristics, cultivation practices, regulatory frameworks, and economic dynamics, focusing on traits aligning with contemporary industrial, medicinal, and market demands.

Combining biomechanics research and investigations into the therapeutic effects of specific substances can facilitate the development of applications utilising different plant parts [27]. For instance, THC, known for its antiemetic and appetite-stimulating properties, has been utilised in approved medications such as Marinol (dronabinol, synthetic THC) and Cesamet to address chemotherapy-induced nausea or vomiting and anorexia associated with AIDS-related weight loss (nabilone, a THC derivative) [27].

Neuroinflammation is a multifaceted response in the brain following injury, involving the activation of glial cells, the release of inflammatory mediators like cytokines and chemokines, and the production of reactive oxygen and nitrogen species [28,29]. Although it is considered a secondary event to neuronal dysfunction or death, neuroinflammation plays a significant role in the onset and progression of neurodegenerative diseases such as Alzheimer’s disease (AD), Parkinson’s disease (PD), multiple sclerosis (MS), Chronic Traumatic Encephalopathy (CTE) [28,29]. Due to the limited efficacy of current treatments for these conditions, neuroinflammation has emerged as a promising therapeutic target in drug discovery [28,29]. Consequently, various in vivo and in vitro models of neuroinflammation have been developed to study its mechanisms and potential interventions. This review aims to provide a comprehensive overview of the current understanding of the interactions and synergistic effects among cannabinoids, terpenes, and flavonoids in cannabis with special focus on their anti-neuroinflammatory and neuroprotective attributes.

## 2. Phytochemicals in Medicinal Cannabis

**Figure 1:**
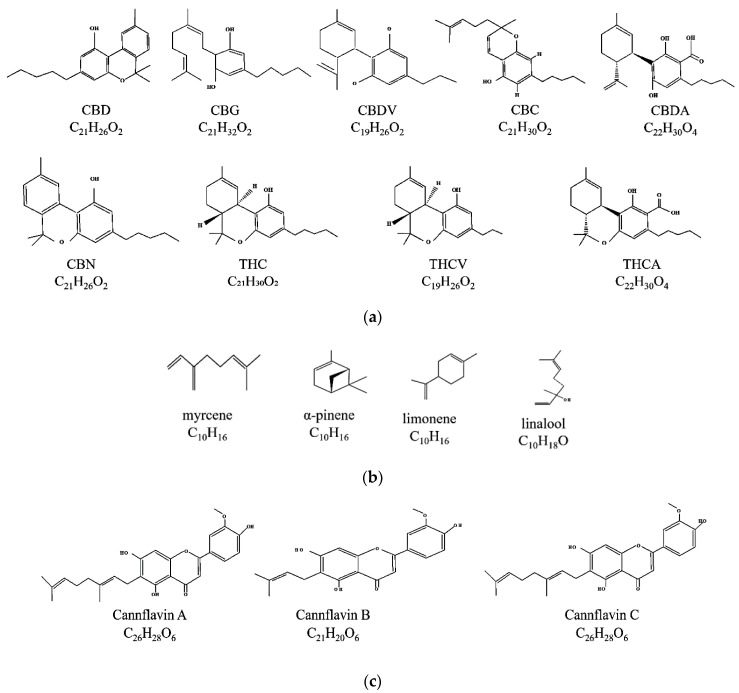
Chemical structures and molecular formula of the main selected (**a**) cannabinoids, (**b**) terpenes, and (**c**) prenylflavonoids in *C. sativa*.

*C. sativa* contains a wide range of phytocannabinoids, which are oxygenated aromatic hydrocarbons derived from meroterpenoids with various substitutions in the resorcinol core (Figure 1) [7,30]. These phytocannabinoids often have alkyl side chains with an odd number of carbon atoms and are initially produced in their acid form (Figure 1). Through decarboxylation, they are converted into their active forms [30]. The two most abundant phytocannabinoids in *C. sativa* are CBDs and THCs (Figure 1). Additionally, cannabigerol (CBG) and its acid form CBGA serve as core intermediates and provide phytocannabinolic acids (Figure 1) [7,31].

Terpenes, which are the second-largest class of cannabis constituents after phytocannabinoids, are also present in *C. sativa* and many other non-cannabinoid plants such as tea, thyme, Spanish sage, and citrus fruits [32]. The major terpenes in *C. sativa* include myrcene, alpha-pinene, linalool, and limonene [32]. In addition, *C. sativa* also biosynthesises flavonoids, including cannflavins, which are prenylated (C5) and geranylated (C10) flavones [33].

## 3. The Endocannabinoid System and Neuroinflammation

Neuroinflammation refers to a broad spectrum of immune responses in the central nervous system that stem from peripheral inflammation [34]. Key cellular players in this process include microglia and astrocytes, which are primary cells involved in the immune reactions within the central nervous system [34]. The activation of a neuroinflammatory response occurs due to peripheral inflammation affecting various components, such as the blood–brain barrier (BBB), glial cells, and neurons [34]. Previously, it was widely believed that BBB, a specialised type of endothelium, ultimately separated the central nervous system from the peripheral immune system [34]. However, it has been discovered that the BBB can become permeable to pro-inflammatory molecules generated during peripheral inflammation and facilitate their release and transport into the brain [35,36]. This neuroinflammatory reaction leads to synaptic dysfunction, neuronal loss, and exacerbation of various brain disorders [37,38,39].

Microglial cells are a crucial component of the central nervous system (CNS) immune defence and maintenance of homeostasis [40,41,42,43]. They act as resident macrophages, responding to pathogenic invasion, tissue damage, and protein aggregates by recognising danger-associated molecular patterns (DAMPs) or pathogen-associated molecular patterns (PAMPs) through specific receptors [42,43,44].

Microglia can migrate to the injury site and initiate an innate immune response when activated [45]. Additionally, they play a critical role in preserving synaptic plasticity and contribute significantly to learning and memory processes by modifying synapses associated with learning [46]. Recent advances in single-cell RNA sequencing have revealed a distinct subtype of microglia known as disease-associated microglia (DAM), which has been implicated in the progression of AD [47]. The blood–brain barrier (BBB), consisting of tight junctions between brain endothelial cells, restricts the entry of pathogenic microorganisms into the CNS. However, certain head injuries or infections can significantly change brain function and behaviour. Inflammatory responses involving pro-inflammatory cytokines are observed when brain tissue is damaged or infected, and microglial activation plays a key role in this process [48].

In neurodegenerative diseases, microglia are associated with neuroinflammation by activating cell surface receptors, such as toll-like receptors (TLRs), scavenger receptors, and the nucleotide-binding domain, leucine-rich-containing family, pyrin domain-containing-3 (NLRP3) inflammasome [49,50,51,52,53]. Impaired microglial phagocytic ability and reduced amyloid-beta (Aβ) clearance are observed in these conditions, characterised by altered expression of Aβ phagocytosis receptors and elevated cytokine levels. The dysregulation of immune receptors, such as TREM2 and CD33, further highlights the significant role of neuroinflammation in neurodegenerative diseases [54,55,56,57,58,59,60,61,62].

**Figure 2:**
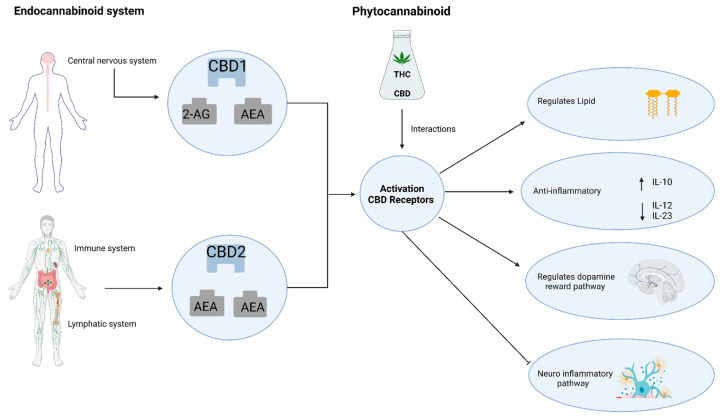
The mechanisms of activating CBD receptors through the endocannabinoid system and phytocannabinoid on neuroinflammation.

THC and CBD are phytocannabinoids in *C. sativa*. They exert their effects on neuroinflammation primarily through activating CB1 and CB2 cannabinoid receptors (Figure 2) [63]. In addition to these receptors, the endocannabinoid system (ECS) includes proteins involved in synthesis, inactivation, and other endocannabinoid molecular targets. Key components of the ECS include endogenous ligands such as arachidonyl ethanolamide (AEA) and 2-arachidonylglycerol (2-AG), which are derivatives of the polyunsaturated fatty acid arachidonic acid [64].

CB1 receptors are predominantly found in the central nervous system, while CB2 receptors are primarily expressed peripherally in lymphoid organs, peripheral blood leukocytes, mast cells, and to a lesser extent in the pancreas [65,66]. CB1 mRNA and protein expression have been observed in various immune cells, including B cells, NK cells, neutrophils, CD8^+^ T cells, monocytes, and CD4^+^ T cells, albeit in decreasing order, whereas CB2 is expressed at higher levels in these immune cells, approximately 10–100 times more than CB1 [67]. Given their widespread expression in the immune system, these receptors may play a crucial role in immunomodulation.

Endocannabinoids, such as 2-AG and AEA, are produced in large quantities by microglia, macrophages, astrocytes, and neurons during inflammation. These endocannabinoids bind to CB receptors and have been shown to reduce neuronal damage by protecting the nervous system from excitotoxicity (Figure 2, Table 1) [68,69,70,71].

Furthermore, cannabinoid treatment has been demonstrated to attenuate the inflammatory effects of IL-1 and protect glial cells from death [72,73]. Overall, the cannabinoid system plays a protective role by combating CNS excitotoxicity and neuroinflammation. The evidence primarily supports the anti-inflammatory benefits of cannabis, although some studies suggest potential pro-inflammatory effects, creating a more nuanced understanding [74,75].

The endocannabinoid system (ECS) modulates multiple physiological processes within the nervous system, and dysregulation of ECS has been associated with various pathological conditions, including neuroinflammation [76,77]. Therapeutic modulation of ECS activity has shown beneficial effects on medical conditions related to neuroinflammation [78,79]. The ECS comprises multiple receptors, including peroxisome proliferator-activated receptors (PPARs) and ion channels (such as the transient receptor potential ankyrin (TRPA) family and the transient receptor potential vanilloid (TRPV) family), as well as cannabinoid receptor types 1 and 2 (CB1 and CB2, respectively) [80]. The ECS also involves endocannabinoids derived from arachidonic acid, receptor ligands, and enzymes responsible for endocannabinoid metabolism [77].

Endocannabinoids, the enzymes involved in their biosynthesis and degradation, and endocannabinoid receptors are expressed by most immune cells [81]. CB1 and CB2 receptors are present in immune cells, with CB2 being expressed at higher levels than CB1 [82,83]. Activation of CB receptors regulates anti-inflammatory responses, as evidenced by an increased release of the anti-inflammatory cytokine IL-10 and a decreased release of pro-inflammatory cytokines IL-12 and IL-23 upon CB2 receptor activation in activated macrophages (Figure 2) [84,85]. The CB2 receptor system has also been implicated in anxiety, depression, and substance abuse, suggesting its involvement in modulating dopamine reward pathways [86,87,88]. Trans-caryophyllene has demonstrated neuroinflammatory inhibition and lipid regulation properties [89].

## 4. Anti-Neuroinflammatory Activity of Phytochemicals in *C. sativa*

### 4.1. CBD

**Figure 3:**
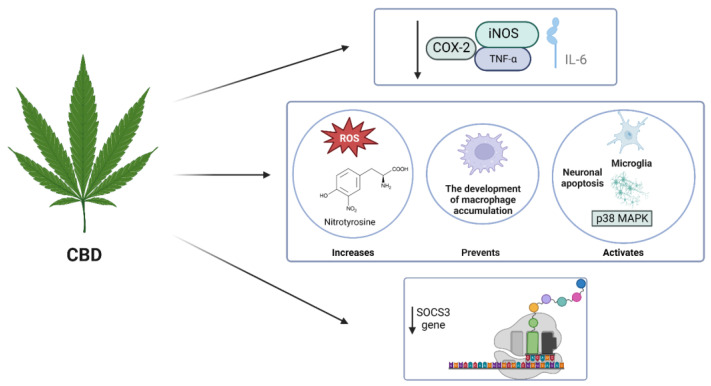
The anti-neuroinflammatory activity of CBD.

CBD has been extensively studied for its potential anti-neuroinflammatory properties in various in vitro and in vivo models of degenerative diseases (Table 1). However, the precise mechanism underlying its anti-neuroinflammatory activity still needs to be understood. In the context of hypoxic–ischemic (HI) immature brains in newborn mice, CBD treatment was found to significantly decrease the expression of inflammatory markers such as IL-6, TNF-α, COX-2, and iNOS in brain slices (Figure 3). It has been suggested that this effect may be mediated through the CB2 and adenosine A2A receptors [90]. Similarly, low doses of CBD were observed to reduce TNF-α production in mice treated with lipopolysaccharides, and this effect was abolished in mice lacking the A2A receptor and restored by an A2A adenosine receptor, indicating a potential modulation of adenosine signalling by CBD [91].

Furthermore, CBD selectively inhibits GPR55, another G-protein-coupled receptor in human macrophages. In microglial cells isolated from the retinas of newborn rats treated with endotoxin or LPS for acute ocular inflammation, CBD treatment inhibited TNF-α production via the p38 MAPK pathway. In rat retinas exposed to LPS, CBD administration prevented the development of macrophage accumulation, activated microglia, increased levels of reactive oxygen species (ROS) and nitrotyrosine, and activated p38 MAPK, and neuronal apoptosis (Figure 3) [92].

In LPS-activated microglial cells (BV-2 cells), CBD has been shown to reduce the production and release of inflammatory cytokines such as IL-1, IL-6, and IFN-β. This reduction is associated with a decrease in the activity of the NF-κB pathway and the levels of IL-1β and IL-6. Additionally, CBD downregulates the expression of the SOCS3 gene, which regulates cytokine and hormone signalling. CBD treatment leads to increased phosphorylation of the STAT3 transcription factor, which is required for activation. In contrast, CBD decreases the phosphorylation of STAT1, a transcription factor involved in IFN-β-dependent pro-inflammatory processes (Carow & Rottenberg, 2014; Kozela et al., 2010). NF-κB and STAT3 have important and sometimes overlapping roles in pro-inflammatory responses, while STAT1 plays a significant role in IFN-β-mediated inflammation [93,94].

### 4.2. THC

Since its synthesis in 1964, THC has been the most extensively studied phytocannabinoid, primarily due to its pharmacological effects. THC primarily interacts with the endocannabinoid receptors CB1 and CB2, acting as a partial agonist at sub-micromolar doses. These receptors have been the focus of considerable research in understanding the psychoactive effects of THC. The development of synthetic high-affinity counterparts has facilitated the identification of the endocannabinoid system and its central nervous system targets [95]. The metabolic precursor of THC, Δ^9^-tetrahydrocannabinolic acid (THCA), is present in high concentrations in cannabis plants. Upon drying or burning, THCA is decarboxylated to THC. THCA is believed to have less psychoactive properties than THC [95]. However, at concentrations exceeding 10 µM, THC inhibits cyclooxygenases-1 and -2, as well as diacylglycerol lipase alpha (DLG), an essential enzyme in the biosynthesis of the endocannabinoid 2-arachidonoylglycerol (2-AG). In vitro, experiments have shown activation of TRPA1 and TRPV4 channels, while TRPM8 channels are blocked at low micromolar concentrations [95].

Another cannabinoid present in varying levels of cannabis is Δ^9^-tetrahydrocannabivarin (THCV). Similar to THC, THCV acts as a partial agonist of CB1/2 receptors and exhibits activity on GPR55, TRPA1, and TRPV1-4 receptors at sub-micromolar or low micromolar doses (Pertwee & Cascio, 2014). In vitro and in vivo animal models have demonstrated the anti-seizure effects of THCV in one study [96].

**Figure 4:**
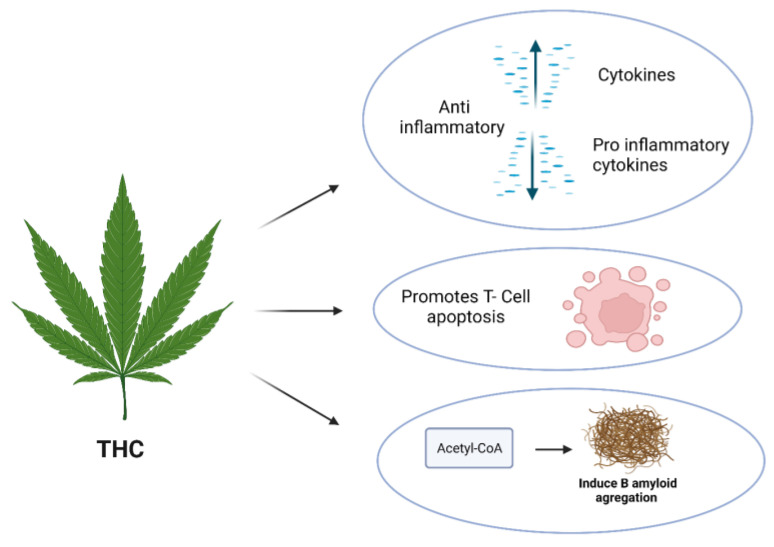
The anti-neuroinflammatory activity of THC.

THC has been shown in numerous studies to possess anti-neuroinflammatory properties (Table 1). For instance, it increases the production of anti-inflammatory cytokines while decreasing pro-inflammatory cytokine production in multiple sclerosis (MS). THC also promotes apoptosis in T cell-driven inflammation and increases the population of FoxP3+ regulatory T cells through miRNA induction and epigenetic modifications (Figure 4) [97,98].

Moreover, THC has been found to inhibit acetylcholine esterase (AchE)-induced aggregation of amyloid-beta (Aβ), improve motor coordination deficits in R6/2 mice, mitigate striatal atrophy and huntingtin aggregate accumulation, and exacerbate malonate lesions in AD (Table 1) [99,100,101,102]. THC, THCA, and the metabolite cannabinol (CBN) have been described to possess analgesic, anti-inflammatory, and neuroprotective effects [103,104,105].

### 4.3. CBG

**Figure 5:**
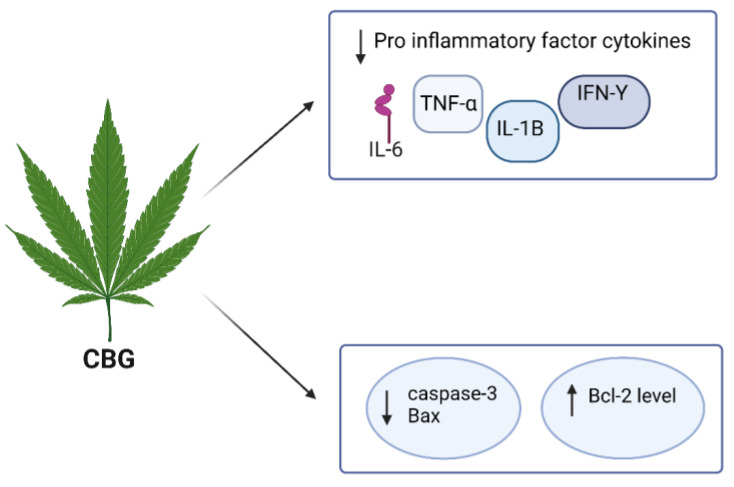
The anti-neuroinflammatory activity of CBG.

While there is still a need for further research on the anti-neuroinflammatory effects of CBG compared to CBD, several studies have discussed the neuroprotective properties of CBG against neuroinflammation (Table 1). For instance, in cultured motor neurons, CBG pre-treatment was found to reduce the levels of pro-inflammatory cytokines such as IL-1β, TNFα, and IFN-γ, and prevent apoptosis in LPS-stimulated macrophages by inhibiting the expression of caspase-3 and Bax, while increasing Bcl-2 levels [106]. Similarly, in an in vivo study using a 3-nitro propionate model to examine the effects of CBG on Huntington’s disease pathology, treatment with CBG significantly attenuated the upregulation of COX-2, iNOS, and pro-inflammatory cytokines such as TNF-α and IL-6 (Figure 5) [107].

These findings highlight the potential of CBG as a neuroprotective agent against neuroinflammation, but further investigation is necessary to understand its mechanisms and therapeutic potential fully.

**Table 1:** A summary of preclinical evidence of cannabinoids on microglial activation and neuroinflammatory signalling.

Compound	Model	Concentration/Dose	Indicated Neurodegenerative Diseases	Outcome	References
CBD	in vitro glutamate neuronal toxicity model	N/A	N/A	CBD was shown to be more protective than either α-tocopherol or vitamin C and comparable to butylated hydroxytoluene (BHT)	[108,109]
THC	in vivo in hemiparkinsonian rats	N/A	PD	neuroprotective effect	[110]
CBD	in vivo in hemiparkinsonian rats	3 mg/kg	PD	exhibited a potent neuroprotective effect in this rat model	[110]
CBD	N/A	<1 μM	N/A	inhibit activated microglial cell migration by antagonising the abnormal cannabidiol (Abn-CBD)-sensitive receptor	[111]
CBD	in vitro PC12 cells	N/A	AD	neuroprotective against the neuronal damage induced by the β-amyloid peptide (Aβ); inhibits Aβ-induced neurotoxicity	[112]
CBD	in vivo mouse model	N/A	AD	attenuated the expression of several glial pro-inflammatory proteins, including glial fibrillary acidic protein, inducible nitric oxide synthase (iNOS), and interleukin 1β (IL-1β), which are major contributors to the propagation of neuroinflammation and oxidative stress	[113]
CBD	in vivo mouse model	100–200 mg/kg	Dravet syndrome	it has beneficial effects on seizures and social deficits	[114]
CBD	in vivo mouse model	10 mg/kg twice daily	Schizophrenia	improves social and cognitive dysfunctions	[115]
CBDV	clinical trial	Single oral dose	ASD	it modulates glutamatergic but not γ-aminobutyric acid (GABA) neurotransmission in adult male patients, although the biological response may differ between autistic individuals	[116]
THCV	in vivo mouse model	<3 mg/kg	PD	alleviates motor inhibition in 6-OHDA-lesioned rodents by blocking CB_1_ receptors at low doses	[117]
THC	N/A	N/A	PD	it reduced levodopa-induced dyskinesia	[118]
CBN	in vitro C6 glioma cells	0.3–30,000 nM EC50: 700 nM	N/A	it inhibited NO production and iNOS expression	[113]
CBN	N/A	N/A	MS	it may antagonise the 2-AG-induced recruitment of microglial cells and produces minimal palliative effect	[111]
THC	in vitro BV-2 murine microglial cell line	10 μM	N/A	it decreases the production and release of pro-inflammatory cytokines, including interleukin-1β, interleukin-6, and interferon (IFN)β, from LPS-activated microglial cells	[94]
CBG	in vitro murine microglial cell line	25 μM	MS	it inhibited the microglia-driven inflammatory response, protected neurons from toxic insults in vitro, and restored motor function impairment by inhibiting the synthesis of IL-1β, IL-6, TNF-α, the chemokine, MIP-1α, and prostaglandin E2 (PGE2)	[119,120]
CBG	in vitro NSC-34 motor neurons	7.5 µM	N/A	CBG pre-treatment REDUCED IL-1β, TNF-α, IFN-γ, and PPARγ protein levels and reduced nitrotyrosine, SOD1, and iNOS protein levels and restored Nrf-2 levels	[106]
CBG	in vivo and in vitro	N/A	PD	it shows a neuroprotective effect against inflammation-driven neuronal damage, acting through the activation of the canonic binding site in PPARγ receptors	[121]
CBG	in vivo and in vitro neuroblastoma Neuro-2a (N2a)	2 g/6.319 mM	HD	it improved motor deficits, reactive astrogliosis, and microglial activation, inhibiting the upregulation of pro-inflammatory markers and improving antioxidant defences in the brain	[122]
CBDA	in vitro Neuro-2a (N2a) cells	25 μM	HD	CBDA shows potent neuroprotective activity by activating PPARγ with higher potency than their decarboxylated products	[123]
CBDA	in vivo	10 and 30 mg/kg	Dravet syndrome	it has an anticonvulsant effect against pentylenetetrazol-induced seizures and hyperthermia-induced seizures	[124]
CBDV	in vivo mouse model	CBDV	Rett syndrome (RTT), a rare neurological disorder affecting predominantly females	it improves behavioural and functional deficits	[125,126,127,128]
CBC	in vitro	1 μM	N/A	CBC exerts potential actions on brain health through effects on adult neural stem cells using whole brain-derived neural stem progenitor cells (NSPCs)	[129]
THC	in vitro	10 μM	N/A	THC reduces IL-1β, IL-6, and TNFα production in LPS-stimulated rat microglial cells	[130]
THC	in vitro	0–15 μM	AD	it inhibits the enzyme acetylcholinesterase (AChE) and prevents AChE-induced amyloid β-peptide (Aβ) aggregation, which is considered the key pathological marker of Alzheimer’s disease	[101]
THC	in vivo R6/1 mouse model	10 mg/kg	HD	it inhibits acetylcholine esterase (AchE)-induced aggregation of Aβ and attenuates the motor coordination deficits of R6/1 mice	[100]
THCA	in vitro N2a cells	10 μM IC_50_ of 0.47 μM	HD	it has neuroprotective activity by activating PPARγ transcriptional activity	[123]

### 4.4. Terpenes 

Terpenes and terpenoids, found in plant resins and essential oils, are significant components responsible for the pharmacological effects of various medicinal plants, including cannabis. Terpenes are hydrocarbons, while terpenoids contain additional functional groups derived from different chemical elements, making them the most abundant class of phytochemicals. In cannabis, there are approximately 200 unique terpenes, focusing on the primary terpenes found in the highest concentrations. These aromatic essential oils contribute to the distinctive aromas, flavours, and characteristics of different cannabis strains [131,132].

Terpenes have lipophilic properties and interact with various bodily targets, including neurotransmitter receptors, ion channels in muscles and neurons, G-protein receptors, enzymes, cell membranes, and second messenger systems. They work independently and synergistically with cannabinoids to produce various therapeutic effects. Additionally, terpenes can enhance the permeability of the blood–brain barrier, leading to the development of transdermal cannabinoid patches containing terpenes as permeation agents. They also influence the binding of THC to CB1 receptors, contributing to the analgesic effects of cannabinoids [103].

While terpenes have been associated with health benefits such as analgesia, anxiolytic and antidepressant effects, skin penetration enhancement, cancer chemoprevention, and antimicrobial activities, their anti-neuroinflammatory activities have not been extensively studied. It is important to note that most available data come from preclinical studies conducted using animal models or in vitro experiments. Some reported benefits of specific terpenes are based on studies evaluating whole essential oils or plants, where the specified terpene may be the most abundant constituent. Additionally, the potential therapeutic contributions of minor terpenes should be considered. Among the primary terpenes found in cannabis are -caryophyllene, myrcene, -pinene, humulene, linalool, limonene, terpinolene, terpineol, ocimene, valencene, and geraniol [133,134].

Myrcene is commonly found in aromatic plants such as sweet basil, bay leaves, lemongrass, and mango. It is utilized in the cosmetic industry due to its remarkable anti-inflammatory, analgesic, and anxiolytic properties [135]. The analgesic effects of myrcene appear to be mediated through an opioid mechanism, as they were inhibited by naloxone [136]. Additionally, myrcene exhibits muscle relaxant, hypnotic, sedative, sleep aid, and antioxidant properties [137].

Alpha-pinene contributes to the distinctive scent of fresh pine needles, conifers, and sage. It is also present in various herbs, including parsley, rosemary, basil, and dill, making it the most prevalent natural terpenes [138]. Studies have demonstrated its antioxidant activity [139] and anti-inflammatory effects in human chondrocytes [140,141], suggesting its potential for anti-osteoarthritic activity [141]. Alpha-pinene also acts as an acetylcholinesterase inhibitor, enhancing memory and counteracting the short-term memory loss caused by THC [142].

Extensive research indicates that linalool, a monoterpene, possesses anti-ischemic, antioxidant, and anti-inflammatory properties. It enhances the activities of antioxidant enzymes superoxide dismutase (SOD) and catalase in vitro, inhibits LPS-induced MCP-1 in airway epithelia, scavenges reactive oxygen species (ROS) in neurons after oxygen–glucose deprivation/reoxygenation, and inhibits MCP-1-induced microglia migration. Linalool also protects neurons from glutamate-induced oxidative stress by preventing mitochondrial ROS and calcium synthesis. Furthermore, it can potentially block LPS-induced PGE2 synthesis and NF-κB/TNF-α expression in macrophages and microglia [143].

Limonene, a monoterpene, exhibits significant anti-inflammatory and antioxidant effects both in vitro and in vivo. It reduces IL-1-induced nitric oxide synthesis in human chondrocytes and decreases the production of prostaglandin E2, nitric oxide, and TNF-α/IL-1 in macrophages stimulated with lipopolysaccharides (LPSs). Moreover, in animal models of colitis, limonene has been shown to alleviate intestinal inflammation when administered in vivo. It also demonstrates nonprotective effects by targeting COX-2 and nitric oxide, preventing renal injury. Additionally, limonene enhances the activity of antioxidant enzymes superoxide dismutase (SOD), catalase, and glutathione in the central nervous system during cerebral ischemia models, while reducing the generation of IL-1 and reactive oxygen species (ROS), thus exhibiting its antioxidant potential [143].

### 4.5. Flavonoids

**Figure 6:**
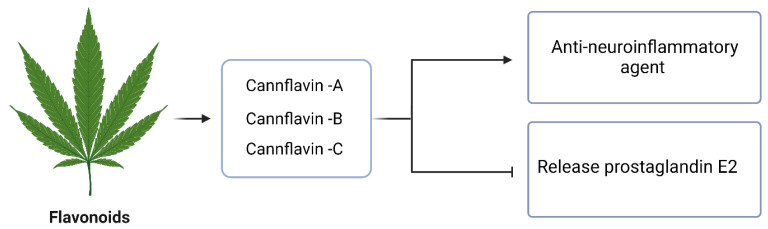
The anti-neuroinflammatory activity of flavonoids in cannabis.

Flavonoids are a class of phenolic compounds characterized by the presence of a phenol ring in their molecular structure. These compounds are known to possess various health benefits, although most of the research conducted so far has been in preclinical models [23]. Among the flavonoids found in cannabis, three cannflavins, namely cannflavin A (CFL-A), B (CFL-B), and C (CFL-C), have been identified. These cannflavins exhibit promising therapeutic properties, particularly as anti-neuroinflammatory agents [144]. In a series of studies conducted in the mid-1980s, Barret et al. investigated the ability of these compounds to inhibit the release of prostaglandin E2 (PGE2) from human rheumatoid synovial cells (Figure 6). The results showed that cannflavins were approximately 30 times more potent than aspirin in ex vivo experiments [144].

## 5. Entourage Effects among the Phytochemicals in *C. sativa*

In 1998, a groundbreaking study conducted by Mechoulam et al. unveiled a pair of monoacylglycerols that influenced the activity of the endogenous cannabinoid 2-arachidonoyl-glycerol through inhibiting its metabolism [145,146]. Despite being pharmacologically inert on their own, these compounds exhibited a significant impact on the activity of the target compound, giving rise to the concept known as the “entourage effect.” This effect refers to modifying the pharmacological properties of individual molecules through interactions with co-existing metabolites, even if these metabolites lack inherent pharmacological activity [147].

Throughout history, cannabis has been utilized as a medicinal plant, and its crude extracts have been found to contain various phytomolecules, such as flavonoids, terpenes, and phytocannabinoids. Recent research has emphasized the preference for combining these phytomolecules in medical therapies due to the observed entourage effect. This phenomenon encompasses two types of interactions: “intra-entourage”, arising from interactions among phytocannabinoids or terpenes, and “inter-entourage”, resulting from interactions between phytocannabinoids and terpenes [148]. Investigating the combinations of phytomolecules exhibiting entourage effects is crucial for developing novel drugs [148].

### 5.1. The Preclinical and Clinical Evidence

Preclinical studies have demonstrated the interaction between phytocannabinoids and terpenes, suggesting that the enhanced medical benefits of full-spectrum cannabis extracts, compared to isolated molecules, can be attributed to the entourage effect [149,150]. However, it is essential to note that unfavourable interactions, referred to as the “parasitage effect”, can also occur in specific in vitro molecular interactions [149].

Careful selection of active phytomolecules and reduction of inactive or potentially pro-inflammatory compounds hold promise for optimizing therapeutic activity. Research has shown that the THCA-rich fraction of a cannabis strain exhibits superior anti-inflammatory activity compared to the crude extract, suggesting the potential benefit of selectively choosing compounds [151].

**Figure 7:**
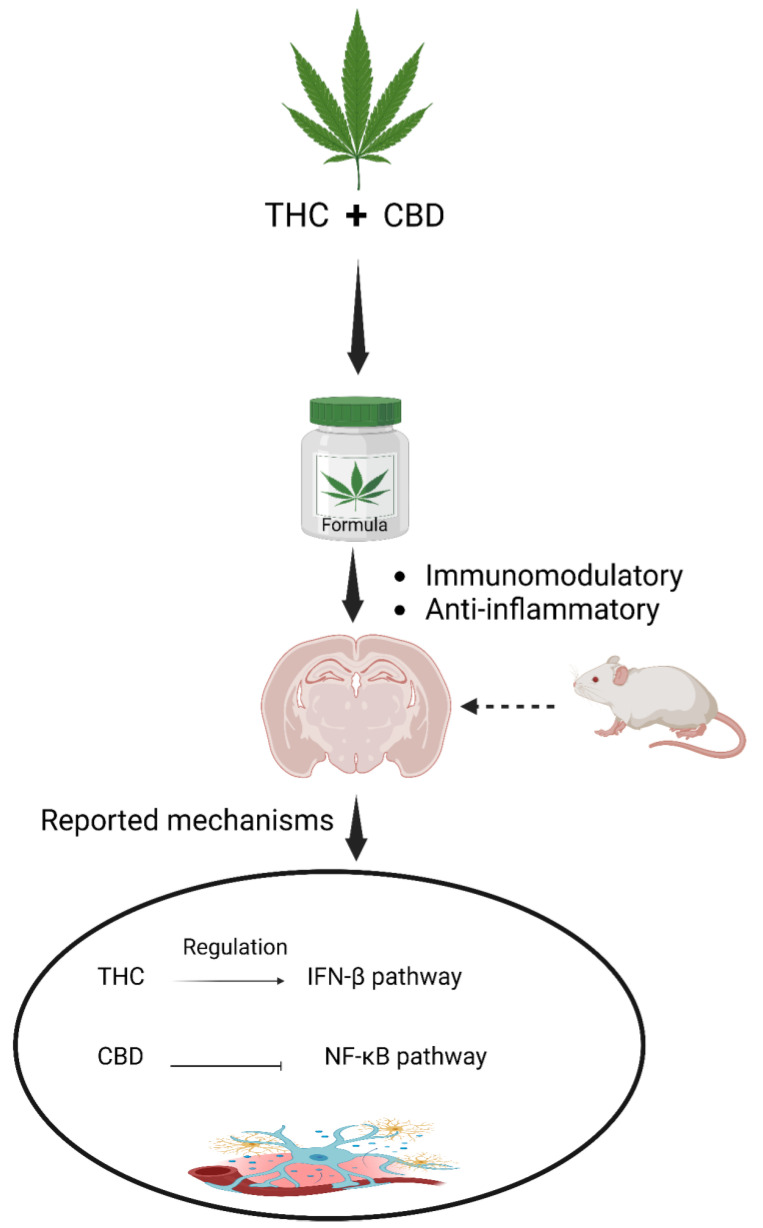
The activity of THC and CBD combination in mediating anti-neuroinflammatory properties.

Moreover, recent studies have demonstrated the suppressive effect of a combination of THC and CBD on neuroinflammation in animal models of multiple sclerosis [152,153]. Phytocannabinoids, including THC and CBD, exhibit immunomodulatory and anti-inflammatory properties, acting through distinct signalling pathways. For example, in LPS-activated microglial cells, THC and CBD were found to exert different mechanisms of action, with THC controlling the IFNβ pathway activity and CBD inhibiting the NF-κB-dependent pathway (Figure 7) [94].

### 5.2. The Entourage Effects in the Context of Neuroinflammation

The entourage effect of cannabis in the context of neuroinflammation and neurodegenerative disorders is a fascinating phenomenon that underscores the complex interplay between various phytochemicals found in *C. sativa* [154]. Extensive research has demonstrated that the therapeutic potential of cannabis extends beyond the individual effects of its primary cannabinoids, such as CBD and THC [155]. Instead, it is the combined action of these cannabinoids, along with a diverse array of terpenes and flavonoids, contributing to the entourage effect, leading to a more comprehensive and robust therapeutic response [155].

The endocannabinoid system (ECS) is central to the impact of the entourage effect on neuroinflammation and neuroprotection (ECS), a crucial physiological system involved in maintaining homeostasis throughout the body [156]. Cannabinoids, such as CBD and THC, interact with the ECS receptors, CB1 and CB2, modulating inflammatory responses and exerting neuroprotective effects [156]. 

In addition to cannabinoids, terpenes play a pivotal role in the entourage effect by enhancing the overall therapeutic potential of cannabis. Terpenes, responsible for the plant’s distinctive aroma and flavour, have been found to possess anti-inflammatory, analgesic, and anxiolytic properties [157,158]. For example, β-caryophyllene, a common terpene in *C. sativa*, has been identified as a selective CB2 receptor agonist with potential anti-inflammatory effects [157]. Moreover, these compounds can influence the blood–brain barrier’s permeability, potentially facilitating the passage of cannabinoids into the brain and central nervous system, where they can exert their neuroprotective effects more effectively.

### 5.3. The Mechanisms That Underpin the Entourage Effects

The therapeutic synergies between phytocannabinoids and various cannabis phytochemicals remain inadequately investigated, with a limited understanding of the underlying mechanisms and pharmacological basis. Santiago et al. (2019) demonstrated that the dominant terpenes in *C. sativa*, namely α-pinene, β-pinene, β-caryophyllene, linalool, limonene, and myrcene, either individually or in combinations, did not impact the hyperpolarization induced by delta-9-THC, suggesting that if phytocannabinoid synergies exist, they do not operate through CB1R or CB2R activation [159]. However, Cheng et al. (2014) reported that β-caryophyllene prefers binding to CB2R, potentially contributing to synergistic effects within the phytochemical matrix of *C. sativa* to mitigate AD-related neurotoxicity [160].

In enhancing bioavailability, the role of terpenoids, particularly their interaction with phytocannabinoids, warrants further exploration. Namdar et al. (2019) highlighted the need for a comprehensive understanding of potential synergistic actions [149]. Terpenes like limonene work through the skin as permeation enhancers for lipophilic compounds. At the same time, linalool demonstrated the ability to improve the permeability of hydrophilic compounds via the same route [161]. Moreover, myrcene’s potential to improve the transportation of delta-9-THC across the blood–brain barrier presents a promising avenue for developing centrally penetrant AD therapeutics [162]. The bioavailability of hydrophobic bioactives, such as phytocannabinoids, is notably lower through ingestion than smoking. Goulle et al. (2008) reported ingestion rates of 6–7%, whereas smoking exhibited higher bioavailability ranging from 10 to 35% [163]. Co-ingestion of triglycerides, particularly long-chain fatty acids, has been identified as a strategy to improve the absorption of ingested lipophilic compounds through the gastrointestinal tract [164]. Additionally, flavonoids, alkaloids, and other polyphenols have revealed a potential bioavailability effect in increasing phytocannabinoids by inhibiting major drug-metabolizing enzymes of the cytochrome P450 family, reducing Phase II metabolism through inhibition of uridine 5′-diphosphate-glucuronosyltransferase, and inhibiting P-glycoprotein 1 efflux pumps [165].

Furthermore, flavonoids, another group of phytochemicals in cannabis, have gained increasing attention for their antioxidative and neuroprotective properties. These compounds have shown promise in combating oxidative stress and neurodegeneration, making them valuable contributors to the entourage effect’s neuroprotective capabilities [161]. A review published in the journal Frontiers in Aging Neuroscience highlighted the neuroprotective effects of various flavonoids, including quercetin and apigenin, which have been shown to attenuate neuroinflammation and reduce neurodegenerative processes [162].

The combined effects of compounds can sometimes result in greater efficacy than the individual constituents. This phenomenon is known as positive potentiating interactions or synergies [163]. In the case of *C. sativa* phytochemicals, the botanical synergies, colloquially known as ‘entourage effects,’ are clinically more effective in vivo and in vitro than a single or predominant phytocannabinoid molecule [164]. Several studies have highlighted beneficial combinations for AD prevention. For instance, in a mouse model of tauopathy, Sativex (1:1 THC/CBD) reduced Aβ and tau deposition in the hippocampus and cerebral cortex [165]. Similarly, a CBD-THC combination in the APPxPS1 mouse model decreased soluble Aβ42 and plaque composition [166]. Another study demonstrated that a combination of CBD and THC may improve cognition in aged transgenic AD mice by normalizing synaptosome-associated protein 25, glutamate receptors 2 and 3, and γ-aminobutyric acid receptor A subunit α1 expression [167]. Schubert and colleagues also demonstrated significant synergistic in vitro enhancement of neuroprotection between delta-9-THC and CBN in an oxytosis cell death assay [168].

Furthermore, studies have shown that *C. sativa*-based extracts, specifically delta-9-THC and CBD-based whole plant extracts, were more effective than the placebo or delta-9-THC-predominant extract for treating cancer pain [169]. Patients with severe epilepsy have also experienced notable improvements with lower CBD extract doses than purified CBD [170]. In mice with seizures induced by pentylenetetrazol, the botanical synergy of minor phytocannabinoids was statistically relevant for treating tonic–clonic seizures and improving survival rates [171]. In non-neurogenic therapeutic areas, *C. sativa* extract treatment was more efficient than pure delta-9-THC in producing antitumor responses in vitro and in vivo [172]. Additionally, humulene was shown to synergize with β-caryophyllene for enhanced anticancer activities [173].

Recent evidence by Finlay and colleagues suggested that terpenoids did not alter the binding of the delta-9-THC, CBD, and CBR radioligand ([3H]-CP55,940) or exert functional effects on CB1R or CB2R, indicating that phytocannabinoid synergies may involve pathways beyond direct effects on these receptors [174].

To further understand entourage pathways, investigations into the effects of terpenoids on cannabinoid metabolism and distribution are warranted, as current studies primarily focus on CB1R and CB2R signalling through the Gi/o protein-coupled receptor pathway [159]. Notably, delta-9-THC may influence signalling at non-cannabinoid receptor targets [175].

Regular consumption of *C. sativa* seeds may elevate endocannabinoid levels due to their high linoleic acid content [11,176], with potential neuroprotective effects explored in preclinical studies [177]. This consumption may also be an absorption enhancer due to the high phytochemical content in seeds, sprouts, and leaves [178]. Cannflavin A, a neuroprotective prenylflavonoid in *C. sativa*, has a prolonged elimination half-life, suggesting that regular hemp sprouts may extend their presence in plasma and tissues [179]. Further studies are needed to explore the potential synergies of whole-plant *C. sativa* extracts in preventing neuroinflammatory diseases.

The significance of the entourage effect in the context of neuroinflammation and neuroprotective disorders offers a novel perspective for developing therapeutic interventions. By harnessing the collective strength of various phytochemicals present in *C. sativa*, researchers and medical practitioners can explore innovative treatment approaches that capitalise on the synergistic interactions of these compounds. Furthermore, understanding the entourage effect can guide the development of targeted cannabis-based formulations tailored to specific neuroinflammatory conditions and neuroprotective disorders, potentially leading to more effective and well-tolerated treatments for those in need.

## 6. Conclusions and Future Directions

In cannabis science, cannabinoids, terpenes, and flavonoids have often been overlooked, with much of the literature focusing predominantly on the major cannabinoids THC and CBD. However, emerging evidence suggests that these constituents, particularly cannabinoids and terpenes, play a substantial role in interacting and collaborating. This interplay gives rise to the diverse effects, benefits, and side effects observed among different cannabis strains, which can vary in the ratios of these components [180]. Moreover, they both interact with the endocannabinoid system and exert various effects on the body, including analgesic, anti-inflammatory, and neuroprotective actions. However, it is becoming increasingly clear that their effects are not solely attributed to their actions but are modulated by other compounds in the plant.

Terpenes, aromatic compounds found in cannabis and other plants, contribute to the distinct flavours and aromas associated with different strains. They have been shown to have pharmacological properties and can interact with neurotransmitter receptors, enzymes, and cell membranes, among other targets. Moreover, terpenes can influence the pharmacokinetics and pharmacodynamics of cannabinoids, potentially enhancing or modulating their effects. The concept of the entourage effect suggests that the combined action of cannabinoids and terpenes may result in a synergistic or additive therapeutic effect greater than the sum of their individual effects.

Flavonoids, another class of compounds found in cannabis, have also demonstrated therapeutic potential. Although research on cannabis flavonoids is limited, studies have suggested their anti-inflammatory, antioxidant, and neuroprotective properties. Furthermore, specific flavonoids, such as cannflavins, have shown potent anti-inflammatory effects, particularly in neuroinflammation.

Understanding the intricate interplay between cannabinoids, terpenes, and flavonoids is paramount for realizing the full therapeutic benefits of cannabis. This paper outlines critical research directions and identifies key evidence gaps necessitating immediate attention.

Firstly, elucidating the synergistic effects and underlying mechanisms of cannabinoids, terpenes, and flavonoids demands a focused investigation.

Secondly, comprehending the intricacies of cannabis phytochemical production and accumulation mechanisms, particularly under varying lighting conditions, is pivotal for advancing medicinal applications.

Thirdly, conducting comprehensive phytochemical characterization of cannabis strains, including their distinct ratios of cannabinoids, terpenes, and flavonoids, holds promise for refining treatment strategies. Such endeavours can pave the way for developing more personalized and productive medicinal interventions.

Moreover, addressing regulatory barriers obstructing cannabis research is imperative. Overcoming these obstacles, stemming from the classification of cannabis as a Schedule I substance, is crucial to expanding access to cannabis products for research purposes. Furthermore, this would enable a more comprehensive exploration of the therapeutic and adverse effects of cannabis and cannabinoids, fostering informed decision making in public health initiatives.

Finally, recognizing the value of non-phytocannabinoid compounds, such as terpenes and flavonoids, in therapeutic development necessitates a broader research focus. Exploring these compounds’ biosynthesis, bioactivities, and biotechnological applications is pivotal for harnessing their therapeutic potential and diversifying treatment options.

In conclusion, a comprehensive exploration of the synergies between cannabinoids, terpenes, and flavonoids, coupled with advancements in phytochemical research and the removal of regulatory barriers, holds the key to unlocking the full therapeutic potential of cannabis. Addressing these gaps is crucial for advancing the field and fostering evidence-based, personalized treatment modalities.

## Data Availability

No new data were created or analyzed in this study. Data sharing is not applicable to this article.
